# The Newer Antiseptics

**Published:** 1897-12

**Authors:** 


### The Newer Antiseptics.

Of the many antiseptic preparations put on the market during the past decade, one of the very best is glyco-thymoline (Kress) put up by the Kress & Owen Co., of New York City. As a cleansing and healing agent in nasal catarrh this preparation stands at the head. Its power to cleanse the nasal tract of all mucus, thus allowing the remedial agent to come in contact with the entire mucous surface, places it at a great advantage over other preparations of this class. The best results are obtained when treatment is begun in the first stage of the disease. The following report by a leading physician of Philadelphia, sets forth very clearly the indications for, and what we may expect from, the use of this remedy.

"GLYCO-THYMOLINE (KRESS) IN NASAL CATARRH.

Nasal catarrh is often a troublesome affection. In its incipiency, apparently a trivial ailment, it may be carelessly treated or entirely neglected. The result is that when a case is brought to the physician's notice it is apt to have assumed a more or less chronic character. Nasal respiration is obstructed by a thick discharge and the presence of crusts.

An antiseptic and unirritant fluid, known as glyco-thymoline (Kress), was recently brought to the writer's notice, and was satisfactorily employed in several cases. The fluid is best applied by means of a Bermingham douche, a cheap and convenient glass apparatus. The glyco-thymoline is diluted by three times or more its bulk of water, according to the age of the patient and the circumstances of the case.

A slender built 11-year old boy, prone to attacks of catarrh of the throat and nose, had suffered for about ten days from a thick and abundant muco purulent discharge from the nose. The nasal passages were coated with thick crusts. Breathing through the nose was impossible, the voice was muffled, and there were frequent attacks of epistaxis. The use of glyco-thymoline, diluted, twice daily, had an excellent effect. Secretion diminished, crusts ceased to form, bleeding soon ceased, respiration became more free, and in a few days the trouble had disappeared. An equally good result was obtained in the case of a child, two years of age, in whom some coryza had been present for about two weeks, and of a lady attacked by an acute coryza, attended by headache and lassitude. A similar experience with several other cases of subacute and chronic nasal catarrh has shown that the local treatment by glyco-thymoline (Kress) is convenient and effective."—G. A. Hewitt, M. D., Philadelphia, in the *Universal Medical Journal*, November, 1896.

Glyco-thymoline is useful in many other conditions, in fact, wherever a good antiseptic preparation is indicated. Its use destroys all odors emanating from the diseased gums and teeth; it restores the alkaline condition of the mouth necessary for the welfare of the teeth, preventing further decay, and keeping the teeth and gums in a healthy condition; it removes all offensive odors from the breath of the tobacco user. Its use internally—a teaspoonful, diluted with water, will give immediate relief from attacks of indigestion with formation of gases in the stomach. When glyco-thymoline is diluted with an equal quantity of water and injected into the rectum, using a small glass or hard rubber syringe, two or three times a day, it is one of the very best palliative treatments for piles, and if this treatment is begun early in the case, it may be curative in its action.

